# Glomerular Endotheliosis in COVID-19-Associated Acute Kidney Injury

**DOI:** 10.7759/cureus.27147

**Published:** 2022-07-22

**Authors:** Barbara Garay, Deep Phachu, Srimathi Manickaratnam

**Affiliations:** 1 Internal Medicine, University of Connecticut Health, Farmington, USA; 2 Nephrology, University of Connecticut Health, Farmington, USA

**Keywords:** proteinuria, glomerular endotheliosis, acute kidney injury, glomerular disorders, covid, endotheliosos

## Abstract

Acute kidney injury (AKI) has been seen in patients hospitalized with a SARS-CoV-2 (COVID-19) infection,but the pathophysiology of glomerular injury is not yet fully understood. We present a case of COVID-19-related “glomerular endotheliosis” in which a 51-year-old female with a 13-year history of class IV lupus nephritis was admitted for COVID-19 pneumonia. Her lupus nephritis had been in complete renal remission for the past 10 years with a baseline serum creatinine level of 1.3 mg/dL and no proteinuria. Her serological workup, including complement levels, was unremarkable. Due to the worsening renal function and persistent proteinuria, she underwent a kidney biopsy that revealed diffuse glomerular endothelial cell swelling, also known as glomerular endotheliosis. Her clinical course unfortunately deteriorated and she succumbed to acute respiratory distress syndrome. As circulating anti-angiogenic factors may contribute to the pathogenesis of endothelial dysfunction leading to glomerular endotheliosis, we propose that a similar circulating antiangiogenic factor may have been triggered by COVID-19 and played a role in our patient’s progressive renal failure.

## Introduction

The incidence of acute kidney injury (AKI) has been reported in as high as 37% of patients hospitalized with a SARS-CoV-2 (COVID-19) infection [1]. Although the exact pathophysiology leading to glomerular injury is not yet fully understood, histologic evaluation of the renal parenchyma of these patients can help shed some light on the mechanism of injury. Several case series have described the morphologic appearance in both kidney biopsies and post-mortem analysis with most demonstrating collapsing glomerulopathy, minimal change disease, membranous glomerulopathy or isolated acute tubular injury [1-4]. Our case presentation is unique in that the electron microscopy revealed “glomerular endotheliosis”, which is characterized by glomerular endothelial swelling with loss of endothelial fenestrae and occlusion of the capillary lumens.

## Case presentation

A 51-year-old morbidly obese Hispanic female with a history of systemic lupus erythematosus complicated by class IV lupus nephritis detected on kidney biopsy done 13 years ago presented to the ED with a one-week history of worsening dry cough and fevers. On admission, temperature was noted to be 102.6 °F and oxygen saturation 88% on room air. Initial lab results revealed a positive SARS-CoV-2 polymerase chain reaction (PCR) test result and a creatinine level of 2.0 mg/dL. There was no noted anemia or thrombocytopenia. Chest x-ray showed a patchy opacity in the left upper lobe and she was admitted for presumed COVID-19 pneumonia.

Her lupus nephritis was in complete renal remission for the past 10 years based on commonly used complete remission criteria with a 24-hour urine collection demonstrating no proteinuria, no hematuria on urinalysis and a stable serum creatinine level of 1.3 mg/dL [5]. Her medications at the time of admission included prednisone 5 mg daily, mycophenolate mofetil 1500 mg daily and hydroxychloroquine 200 mg daily for extrarenal manifestations. Other pertinent medical history included hypertension, and chronic kidney disease stage 3A with an estimated glomerular filtration rate (eGFR) of 44 mL/min/1.73 m^2^ (Chronic Kidney Disease Epidemiology Collaboration, or CKD-EPI).

Upon admission, urinalysis revealed 2+ protein, 4-7 red blood cells per high power field, and no urinary white blood cells. The 24-hour urine collection demonstrated 8.7 g of proteinuria. Serology was unremarkable with a C3 of 169 mg/dL (reference range 67-154 mg/dL) and C4 of 59 mg/dL (reference range 16-66 mg/dL) and double-stranded DNA antibody levels at <12 IU/mL (reference range 16-66 <30 IU/mL). Testing was also negative for lupus anticoagulant dRVVT, IgG/IgM beta-2-glycoprotein 1 antibodies, cardiolipin antibodies, hepatitis B, hepatitis C, HIV and anti-phospholipase A2 receptor antibodies. She was treated with 250 mg solumedrol for three days followed by 60 mg of oral prednisone daily. She was continued on hydroxychloroquine and her mycophenolate was discontinued. No additional COVID-19-specific therapy was given. Given her nephrotic-range proteinuria and worsening renal function, a decision was made to pursue a kidney biopsy.

**Figure 1:**
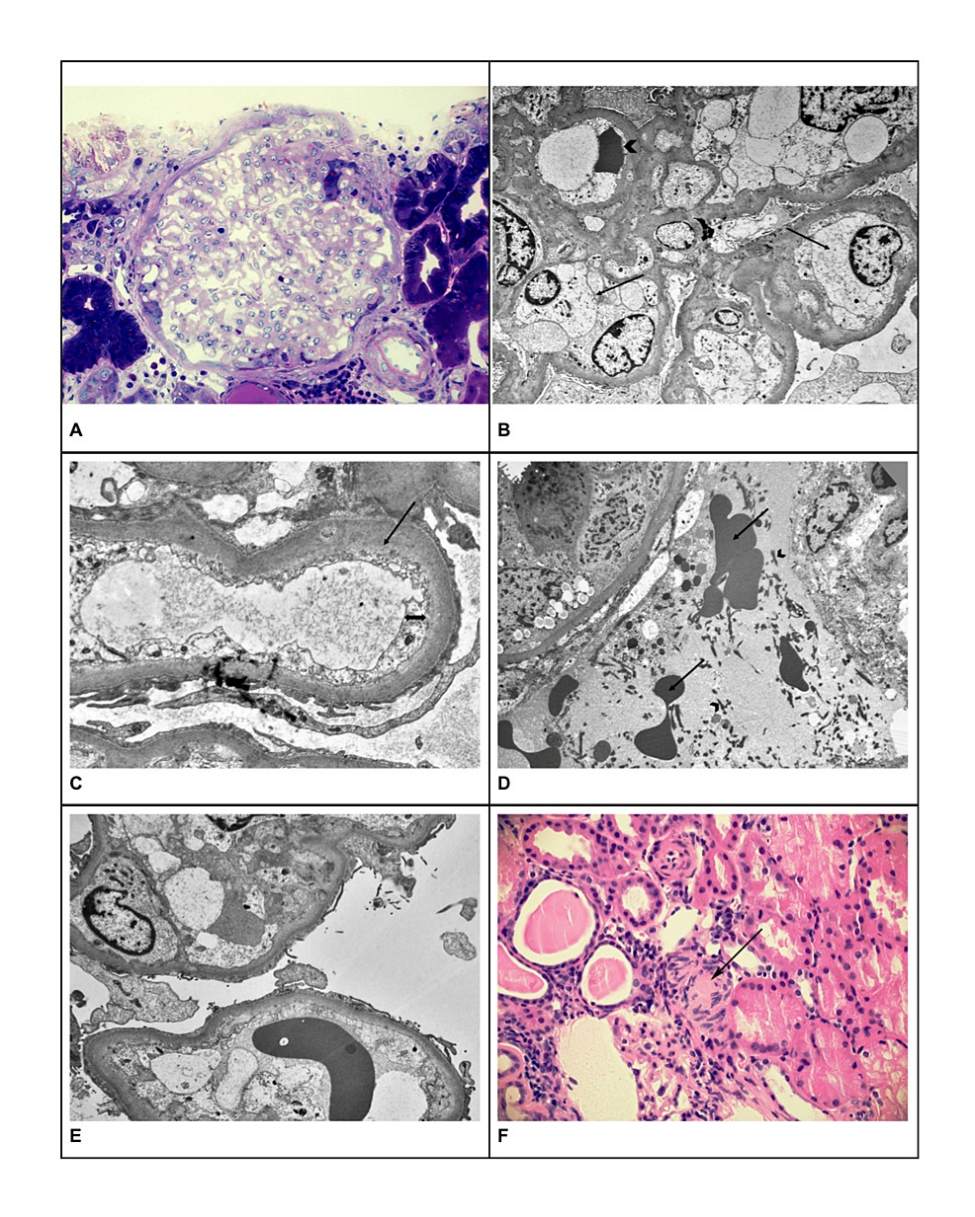
Biopsy images The electron microscopy thick section (A) showing bloodless glomerulus, (B) glomerular capillary loops with edematous endothelial cells (arrows) compressing a passing red blood cell (arrow head), (C) glomerular capillary (arrow) with edematous endothelial cells cytoplasm (double-head arrow), (D) intertubular capillary space with numerous fibrin tactoids (arrow heads) and red cells (arrows), (E) glomerular capillary loops with visceral epithelial cells, (F) arteriole with thrombus (arrow).

Renal biopsy light microscopy demonstrated glomerular compartment sclerosis with subsegmental scleroses without evidence of collapsing focal segmental glomerulosclerosis. There were no mesangial, subendothelial, or subepithelial immune complex-type dense deposits to suggest lupus activity. Electron microscopy (EM) showed diffuse glomerular endothelial cell swelling (endotheliosis) (Figures 1A-1C). EM also revealed tubular capillaries with luminal fibrin tactoids, tubular atrophy/interstitial fibrosis, and diffuse foot process effacement (Figures 1D, 1E). There was also evidence of arteriolonephrosclerosis along with the presence of luminal thrombi (Figure 1F). A viral stain was not available.

Patient’s clinical course unfortunately worsened with multi-organ failure including respiratory distress needing mechanical ventilation and renal failure requiring renal replacement therapy. She subsequently succumbed to acute respiratory distress syndrome.

## Discussion

The mechanism of injury in COVID-19-related AKI is thought to be either directly or indirectly related to excess cytokine production triggered by the viral infection [6]. A recent meta-analysis demonstrated that the prevalence of AKI among hospitalized patients with COVID-19 was 28%, and 46% among the critically ill patients [7]. The prevalence was around 20% in patients needing renal replacement therapy. A multi-center retrospective analysis of 240 kidney biopsies in patients with COVID-19 showed that over 87% of biopsies revealed either tubular injury, podocyte injury, basement membrane thickening, or focal segmental glomerulosclerosis, and thrombotic microangiopathy was seen in only 2.1% of cases [8]. As such, our case is unique as our literature search did not reveal other described cases of isolated glomerular endotheliosis.

Glomerular endotheliosis refers to a morphological disorder characterized by glomerular endothelial edema leading to a reduction in capillary lumen size [9]. The separation of the endothelial cells from the glomerular basement membrane initiates complement activation and can lead to microthrombi formation resulting in thrombotic microangiopathy. Endotheliosis has mostly been reported in cases of preeclampsia, but also been reported in association with parvovirus B19 infection and POEMS syndrome (referring to polyneuropathy, organomegaly, endocrinopathy, monoclonal gammopathy, and skin changes) [9].

The current literature suggests that circulating anti-angiogenic factors contribute to the pathogenesis of endothelial dysfunction and microangiopathy in preeclampsia [10]. One such factor is thought to be soluble fms-like tyrosine kinase 1, which has been detected at higher levels in the plasma of pregnant females with preeclampsia and is known to bind to vascular endothelial growth factor leading to an imbalance between pro-angiogenic and anti-angiogenic factors resulting in endothelial cell dysfunction [11]. When this endothelial dysfunction occurs in the glomeruli, it leads to ballooning of endothelial cells, known as “endotheliosis”, with subsequent occlusion of capillary lumens resulting in renal dysfunction [9,11]. The damaged endothelial cells release endothelin-1 that results in podocyte cytoskeletal dysfunction and podocyte injury leading to proteinuria [10,12,13]. The recent literature has also demonstrated elevated markers of angiogenesis and endothelial injury in patients with COVID-19 infection [14]. Given that there are histological similarities between the endotheliosis seen in preeclamptic patients and our patient, it raises the hypothesis that a similar circulating antiangiogenic factor whose production was triggered by the excessive immunological response to COVID-19 may have played a role in our patient’s progressive renal failure.

## Conclusions

Our case demonstrates that acute kidney injury in COVID-19 can present with a pattern of renal injury similar to the pattern of injury seen in preeclamptic patients. As such, we raise the question whether circulating antiangiogenic factors play a role in this form of kidney injury and whether treatment with plasmapheresis would be beneficial. However, further studies evaluating both serum antiangiogenic factors and the role of plasmapheresis in COVID-19-associated AKI are needed.
